# Glycans as Regulatory Elements of the Insulin/IGF System: Impact in Cancer Progression

**DOI:** 10.3390/ijms18091921

**Published:** 2017-09-07

**Authors:** Julio Cesar M. de-Freitas-Junior, Jéssica Andrade-da-Costa, Mariana Costa Silva, Salomé S. Pinho

**Affiliations:** 1 Cellular and Molecular Oncobiology Program, Brazilian National Cancer Institute (INCA), Rio de Janeiro 20231-050, Brazil; jcjunior@inca.gov.br (J.C.M.d.-F.-J.); jessica_andradecos@hotmail.com (J.A.-d.-C.); 2 Institute of Molecular Pathology and Immunology of University of Porto (IPATIMUP) & Institute for Research and Innovation in Health (i3S), University of Porto, 4200-135 Porto, Portugal; mcsilva@ipatimup.pt; 3 Faculty of Sciences, University of Porto, 4169-007 Porto, Portugal; 4 Faculty of Medicine, University of Porto, 4200-319 Porto, Portugal

**Keywords:** insulin/IGF system, glycans, cancer, glycosylation

## Abstract

The insulin/insulin-like growth factor (IGF) system in mammals comprises a dynamic network of proteins that modulate several biological processes such as development, cell growth, metabolism, and aging. Dysregulation of the insulin/IGF system has major implications for several pathological conditions such as diabetes and cancer. Metabolic changes also culminate in aberrant glycosylation, which has been highlighted as a hallmark of cancer. Changes in glycosylation regulate every pathophysiological step of cancer progression including tumour cell-cell dissociation, cell migration, cell signaling and metastasis. This review discusses how the insulin/IGF system integrates with glycosylation alterations and impacts on cell behaviour, metabolism and drug resistance in cancer.

## 1. Introduction

The insulin/insulin-like growth factor (IGF) system is known to be highly implicated in the control of glucose metabolism and glycaemia, also playing an important role in cell growth, cell differentiation and metabolic pathways [1].

In tumour progression, several studies have demonstrated the impact of the insulin/IGF system on cancer cell behaviour, particularly on the acquisition of the malignant phenotype by regulating the epithelial-mesenchymal transition (EMT) program [2,3,4,5]. Moreover, the insulin/IGF system has also been implicated in cancer metabolism, the acquisition of cancer drug resistance, as well as with cancer stem cell (CSC) phenotypes [6,7], which altogether highlight the importance of this system in the regulatory networks that occur during the processes of cancer development and progression.

Glycosylation is a frequent post-translational mechanism that is characterized by the addition of glycan structures (carbohydrates/sugar chains) to proteins/lipids through the synchronized action of different glycosyltransferases enzymes that act in a stepwise manner in the ER (endoplasmic reticulum)/Golgi compartment of essentially all cells. Glycans have been described as having a relevant impact on both homeostatic and pathological conditions such as cancer. The repertoire of glycans structures expressed in cells change during the transition from normal to malignant phenotypes as a response to genetic and/or environmental stimuli. The expression of aberrant glycans structures in cancer cells has been shown to play an instrumental role in each pathophysiological step of cancer development and progression [8].This review describes how glycans integrate with the insulin/IGF system that include the specific ligands, receptors and signaling pathways, addressing how this biological network affects and controls cell behaviour, drug resistance and metabolism in cancer.

## 2. The Insulin/Insulin-Like Growth Factor (IGF) System

### 2.1. Ligands and Binding Proteins

The insulin/IGF system has several components, including ligands, binding proteins, receptors and downstream proteins. Insulin is a key hormone produced by pancreatic β-cells that controls glycaemia and glucose uptake in several tissues, acting also on lipid synthesis in the liver [1]. The IGFs (IGF1 and IGF2) are peptides structurally similar to insulin, being produced in the liver and in tissues where they act in both the autocrine and paracrine pathways. The IGFs are important mediators of cell growth, differentiation and metabolism. In addition to IGF and insulin, which are responsible for triggering the signaling cascade, there is also a family of six high-affinity IGF-binding proteins (IGFBPs) that modulate the interaction between these ligands and their receptors. In biological fluids, the IGFs are frequently associated with IGFBPs and are rarely found (<1%) in their free form [9,10]. The IGFBPs are found in both precursor and mature form and their molecular structure consists of three domains (amino terminal, central, and carboxyl terminal). The central domain, also known as the binding domain, usually undergoes post-translational modifications such as glycosylation or phosphorylation [11]. IGFBPs play a key role in the availability of ligands through the regulation of their half-life, blocking the interaction with the receptor or even favoring signaling activation due to controlled release of the ligands [12,13]. Evidence has also suggested that IGFBPs may have insulin/IGF-independent functions, including during tumour progression [14].

### 2.2. Receptors

The three main components of the insulin/IGF system are the IGF1 receptor (IGF1R), IGF2 receptor (IGF2R), and insulin receptor (INSR). Splicing variants can also give rise to two additional isoforms of INSR—the INSR-A (short isoform) and INSR-B (long isoform) [15]. The interaction of the isoforms with the IGF1R can also generate the hybrid receptors A (HR-A) and B (HR-B). With the exception of IGF2R, which has a monomeric structure, the other five receptors form a heterotetrameric structure composed of two α and two β subunits. The α subunit is extracellular and responsible for ligand binding, whereas the β subunit has a transmembrane and an intracellular segment where the tyrosine kinase domain is located. The IGF1R autophosphorylation follows binding of the ligands and, for kinase activation, the phosphorylation of three major tyrosine residues (Tyr^1131^, Tyr^1135^ and Tyr^1136^) are needed [16]. 

Phosphorylation in an equivalent tyrosine cluster (Tyr^1146^, Tyr^1150^, Tyr^1151^) is required for the full activation of INSRs [17]. These receptors can be modulated by post-translational modifications in the subunits, in which the α subunit may undergo *N*-glycosylation whereas the β subunit can be modified by both *N*- and *O*-glycosylation, with the latter being described only in INSR [18,19,20].The INSR ectodomain is highly glycosylated, containing in each monomer a total of 19 potential *N*-glycosylation sites of which 14 were found to be occupied by glycan moieties [18]. In adipocytes, glucose deprivation was described to lead to the expression of an aberrantly glycosylated form of the INSR, which prevents oligomerization modulating the insulin-dependent tyrosine kinase activity [21]. Furthermore, *N*-glycosylation of INSR at Asn^397^ or Asn^418^ was described as essential for its normal biosynthesis and processing [22]. Concerning the IGFRs, the α-subunit region of IGF1R also contains 11 potential *N*-glycosylation sites [23], while the extracellular domain of IGF2R exhibits 19 putative sites [24]. In addition, at least 6 mucin-type *O*-glycosylation sites were described in the INSR ectodomain [19]. These glycosylation modifications affect the receptor´s folding, activity and function and may vary accordingly with physiopathological conditions. As an example, changes in the glycosylation of placental INSR and IGF1R have been observed between the first and third trimesters of gestation in pregnant healthy women, including the decrease of fucosylation and α2,6-sialylation of INSR and IGF1R, and an increase of total fucosylation of IGF2R [25].

### 2.3. Downstream Proteins

**Figure 1:**
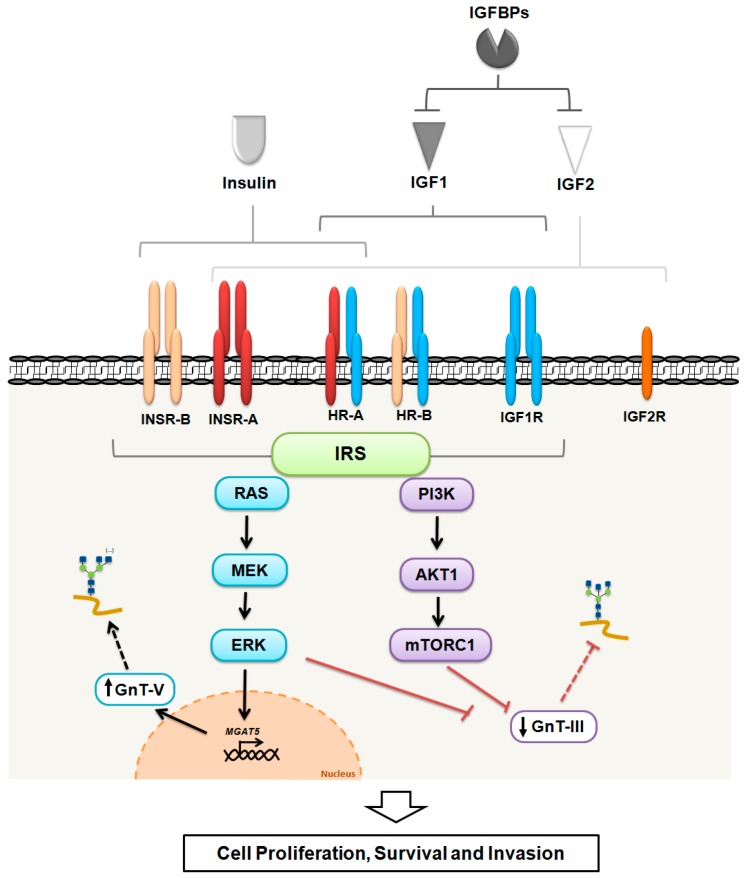
The insulin/insulin-like growth factor (IGF) system. The ligands (insulin, IGF1 and IGF2) bind to their specific receptors triggering downstream signaling pathways (RAS/MEK/ERK and PI3K/AKT1/mTORC1) through IRS proteins, with the exception of IGFR2 that does not transduce signaling. The IGFBPs negatively modulate both IGF1 and IGF2. The activation of RAS/MEK/ERK pathway has been shown to be involved in the upregulation of *MGAT5* gene, increasing the levels of expression of β1,6-branched *N*-glycans. On the contrary, the activation of PI3K/AKT/mTOR signaling cascade was associated with the impairment of GnT-III-mediated bisecting GlcNAc *N*-glycans expression. These signaling pathways have an impact on cell growth, survival and invasion, which favors tumour development and progression. GnT-III: *N*-acetylglucosaminyltransferase III; GnT-V: *N*-acetylglucosaminyltransferase V. GnT-III catalyzes the bisecting GlcNAc *N*-glycan structure and GnT-V catalyzes the β1,6 GlcNAc branched *N*-glycans.

The activation of the insulin/IGF receptors triggers intracellular signaling through insulin receptor substrate (IRS) proteins, a family of adaptor molecules consisting of four closely related members (IRS1–IRS4) and two distant relatives (IRS5 and IRS6, also known as docking proteins (DOK4 and DOK5 respectively)) [26], which connect receptors’ activation to downstream kinase cascades, such as the RAS/MEK/ERK or PI3K/AKT1 pathways. IRS proteins have two ubiquitously expressed forms in humans, IRS1 and IRS2, which are highly regulated by both phosphorylation (Tyr, Ser and Thr) and ubiquitination [27,28]. The RAS/MEK/ERK and PI3K/AKT1 pathways, although they are generally associated with proliferation and survival respectively, are also interconnected to modulate several cellular mechanisms involved in tumour development and progression [29]. Although IRS1 and IRS2 structures are quite similar, the triggered signaling mechanisms differ from each other. Mice lacking IRS1 show insulin resistance and growth retardation, but do not develop diabetes because hyperinsulinemia possibly compensates for this resistance, whereas the disruption of IRS2 impairs insulin secretion by the pancreatic β-cells, causing type 2 diabetes [30,31]. The *IRS3* is a pseudogene in humans [32] and IRS4 induces constitutive PI3K/AKT1 pathway hyperactivation in breast cancer cells [26]. The key elements composing the insulin/IGF system, integrated with glycans alterations and the cellular outcomes, are depicted in Figure 1.

## 3. Impact of the Insulin/IGF System in Cancer Development and Progression

### 3.1. Cellular Behaviour

Several mechanisms can lead to an imbalance of the insulin/IGF signaling network in cancer, including the increased bioavailability of the ligands, dysregulation of signaling proteins, and overexpression of the receptors [33]. Recently, an increasingly number of studies have revealed that insulin/IGF signaling is involved in the acquisition of the malignant phenotype by regulating epithelial-mesenchymal transition (EMT) program, with results in a negative impact on proliferation, invasion, migration, and apoptosis [2,3,4,5].

The EMT, involving the loss of cell-cell adhesion, mainly results in the acquisition of a migratory and invasive phenotype of cancer cells that accompany tumour progression. During this biological process the transcription factors ZEB1 and ZEB2 play a crucial role by binding to E-boxes of *CDH1* (E-cadherin gene) [34]. In prostate cancer cells that have the epithelial phenotype, stimulation with IGF1 upregulates ZEB1 expression in both mRNA and protein levels, leading to E-cadherin repression and upregulation of Fibronectin and *N*-cadherin [35]. Increased levels of Snail, another transcription factor that inhibits E-cadherin expression, was found to be induced by IGF1 in non-small cell lung cancer (NSCLC) cells, promoting EMT [36].

Additionally, evidence has demonstrated that the EMT process gives rise to cells with stemness features (the so-called cancer stem cells, CSC) which contribute to metastasis and drug resistance [37,38,39]. In breast cancer cells, the inhibition of the PI3K/AKT1/mTORC1 pathway or knockdown of IGF1R suppresses the EMT program, reducing stem cell niches [40]. Using thyrospheres models, it was observed that the thyrospheres derived from thyroid cancer have a remarkable increase of INSR-A and IGF2 when compared to normal thyrospheres. However, only IGF2 contributes to the self-renewal process, whereas the increase in the degree of differentiation was associated with the downregulation of the insulin and IGF receptors [41]. In breast cancer cells, a positive feedback mechanism was demonstrated when IGF2 binds to IGF1 receptors, triggering PI3K/AKT1 signaling and leading to the activation of DNA-binding protein inhibitor ID1, a transcriptional factor that acts not only on the maintenance of stemness but also on the positive regulation of IGF2 [42]. Interesting conclusions were also drawn in models of hepatocarcinoma in which the inhibition of Nanog-positive cells, identified to be associated with CSC resulted in down-regulation of IGF1R, influencing the self-renewal capacity of these cells. In addition, the overexpression of Nanog in Nanog-negative cells increase the expression of IGF1R, and the specific inhibition of IGF1R signaling significantly inhibit self-renewal and Nanog expression [43].

Changes in the insulin/IGF system can also be involved in the acquisition of the aggressive cancer phenotype. In hepatocellular carcinoma (HCC), decreased expression of IGFBP1 is correlated with microvascular invasion and metastasis [44]. In patients with gastric cancer, elevated IGF1R levels were associated with lymph node metastasis [45]. Interestingly, in triple-negative breast cancer cells it was also shown that overexpression of IGF1R induces migratory and invasive behaviours in a mechanism mediated by the activation of the focal adhesion kinase (FAK) signaling cascade, which can be suppressed using pharmacological inhibitors of FAKs [46].

The modulation of IGF1R signaling was further found to affect cell death programming through interplay with transforming growth factor β receptor (TGFβR) signaling. Abrogation of TGFβ/Smad3 signaling leads to increased expression and phosphorylation levels of IRS1, resulting in decreased apoptosis by increasing XIAP expression levels in FET human colon cancer cells [5]. Moreover, in triple-negative breast cancer cells the IGF1 increases the cell growth and confers a protective effect against staurosporine-induced programmed cell death [47]. Interestingly, the inhibition of *N*-glycosylation using inhibitors of *N*-glycans biosynthesis resulted in a remarkable decrease of IGF1R autophosphorylation together with its reduced expression at the cell surface, which was accompanied by a substantial decrease in the survival of Ewing’s sarcoma cell lines [48].

Recently, increasing amounts of evidence have shown that microRNA may regulate insulin/IGF signaling. Increased miR-29a expression cooperates with insulin to promote the proliferation of breast cancer cells by increasing ERK phosphorylation [49]. In addition, it has been demonstrated that MicroRNA-30a, through a Src-dependent mechanism, was found to be involved in IGF1-Induced EMT in nasopharyngeal carcinoma cells [50]. In gastric cancer cells it has been shown that IGF1 is able to induce EMT through the up-regulation of ZEB2, in addition, AKT1/ERK inhibitors revert IGF1-induced EMT through up-regulation of miR-200c, suggesting the involvement of an AKT1/ERK-miR-200c-ZEB2 axis in EMT induced by IGF1 stimulation [45].

Interestingly, the integrative microRNA-insulin/IGF regulatory network seems to represent an attractive strategy for the molecular stratification of glioblastoma multiform tumours, since patients who present concomitantly low IGF1 and high miR-181d levels have a significantly longer survival rate than those with high-IGF1 and low-miR-181d [51].

### 3.2. Drug Resistance

Changes in the insulin/IGF system also contribute to the acquisition of resistance to chemo-, radio- and target-therapy [6,7]. A study using chemoresistant colorectal cancer cells, obtained by treatment selection with 5-fluorouracil or oxaliplatin, showed that these cells have a higher expression of CSC markers concomitantly with increased expression and activation of IGF1R. Interestingly, these cells were described to display approximately 5-fold increased responsiveness to treatment with IGF1R inhibitory monoclonal antibody compared to parental cells [52].

In ovarian cancer, the upregulation of IGF1R was associated with the early acquisition of resistance to cisplatin-paclitaxel treatment (single or in combination). In these cells, treatment with IGF1R inhibitor (in combination with cisplatin, paclitaxel or both) was able to reverse the therapy resistance at early stages [53]. Furthermore, it was also shown that the inhibition of IGF1R at early stages of therapy resistance and AKT1 inhibition at late stages were able to abrogate the CSC phenotype. Together, these data demonstrate that the IGF1R/AKT1 signaling pathway significantly impacts the acquisition of chemoresistance in cancer cells.

Experiments carried out using NSCLC cells demonstrated that the induction of EMT leads to resistance to tyrosine kinase inhibitors, however, the silencing of IGF1R (siRNA) in these cells restore their sensitivity to gefitinib or erlotinib [36].

Importantly, clinical studies in gastric cancer patients human epidermal growth factor receptor HER2+ but non-responders to HER2-targeted therapy (including lapatinib) [54], showed that IGF1R and INSR contribute to the acquisition of the resistant phenotype by precluding the lapatinib-induced suppression of cell motility and apoptosis by re-stimulating both AKT1 and/or ERK signaling but also EMT-related signaling [55].

In cancer cells, the absence of IGF1R-linked glycans at Asn^913^ compromised its membranous localization, being associated with insensitivity to figitumumab (a humanized anti-IGF1R antibody), suggesting that changes in the pattern of the expression of *N*-glycans attached to the growth receptor modulate the sensitivity to target therapy in cancer cells [56]. Although these results support the importance of glycans interplaying with the insulin/IGF system in cancer, further studies are required on this topic.

In addition to the mechanisms related to chemoresistance, changes in the insulin/IGF system are also involved in radioresistance. In lung cancer cells, radiation increases IGF1R expression thus triggering a downstream mechanism that leads to repression of p53-induced apoptosis through enhancement of phosphorylation of histone deacetylase-1 (HDAC1), which binds to the p53 promoter [57].

The analysis of glioma stem cells further demonstrated that fractionated radiation promotes both an increase in IGF1 secretion and a gradual upregulation of the IGF1R, which confer radioprotective effects on resistant cells. Interestingly, the treatment of tumours formed by this radioresistant glioma stem cells with picropodophyllin (an IGF1R inhibitor) increased the radiosensitivity [58].

Interestingly, the increased activity of ST6GAL1 (ST6 β-galactoside α-2,6-sialyltransferase 1) that results in increased α2,6 sialylation appears to be involved in the radiation-dependent cell migration of carcinoma cells [59]. Accordingly, we have reported that the inhibition of *N*-glycan biosynthesis was associated with radiosensitization of undifferentiated human colorectal carcinoma HCT-116 cells [60]. However, although other evidence supports that the inhibition of *N*-glycan biosynthesis enhances the effects of radiation in cancer cells [61], it remains unclear how changes in INSR/IGF1R-linked *N*-glycans could interfere with radiosensitivity in cancer cells.

Taken together, and given the impact of the insulin/IGF pathway in cancer drugs resistance mechanisms, the modulation of this pathway might be an attractive strategy to reverse cancer therapy resistance in various types of cancer.

### 3.3. Cell Metabolism

The insulin/IGF system is closely related to cell metabolism. Tumour cells exhibit increased glucose uptake and most of them convert glucose to lactate even in the presence of oxygen (“aerobic glycolysis” or Warburg effect), which constitutes an advantage for growth being considered a metabolic hallmark of cancer [62]. Some of these advantages include: (1) resistance to fluctuation in oxygen tension; (2) production of lactic and bicarbonic acids that favor cell invasion suppressing the immune response; (3) protection against reactive oxygen species through the generation of NADPH; and, importantly; (4) the use of intermediates of the glycolytic pathway to fuel anabolic reactions, such as hexosamine, glycogen, ribose 5-phosphate, triacylglyceride, phospholipid, alanine and malate synthetic pathways [63]. In the case of hypoxic conditions, which is frequently found in solid tumours, cancer cells develop an adaptive program by increasing hypoxia inducible factor 1α (HIF1A), leading to increased expression of both glucose transporters (e.g., Glucose Transporter type 1, GLUT1) and key glycolytic enzymes (e.g., hexokinase (HK) and lactate dehydrogenase (LDH)) [1,64]. Activation of the PI3K/AKT1/mTORC1 signaling pathway under aerobic conditions also contributes to increased levels of HIF1A, thus generating metabolic reprogramming [65]. Accordingly, IGF1 was described to be involved in both the activation of HIF1A and in the expression of GLUT through PI3K/AKT1/mTOR signaling pathway [66,67].

Interestingly, increased HIF1A level seems to be involved in the activation of EMT under hypoxia conditions. In NSCLC cells, hypoxia-induced EMT is accompanied by an increase of IGF1, IGF1R, and IGFBP3, whereas the treatment with AEW541 (IGF1R inhibitor) reverses hypoxia-induced EMT, and the inhibition of HIF1A with YC-1 inhibitor abolishes the activation of IGF1R and reduces the expression of IGF1 and IGFBP3 in hypoxic cells [68].

Epidemiological studies have shown that the risk of developing malignant neoplasm is higher in obese or diabetic individuals, especially for those cancers whose cells exhibit aerobic glycolysis [69,70]. Consistently, a high glucose level itself induces EMT in A549 human lung carcinoma cells [71]. Hyperglycemic conditions also increase proliferation rate of several cancer cells, and this effect may be amplified when in combination with high insulin or IGF1 levels [72,73,74]. Paradoxically, mouse model studies have shown that genetic alterations leading to constitutive activation of the PI3K/AKT1/mTORC1 signaling pathway may promote self-sufficiency in tumour growth [75].

The insulin/IGF network is also highly affected by post-translational modifications through glycosylation. High uptake of glucose in tumour cells leads to increased levels of intracellular fructose-6-phosphate, thus fueling (with ~2–5% of a cell’s glucose) the hexosamine biosynthetic pathway (HBP) by generating substrates (i.e., UDP-GlcNAc, UDP-GalNAc, CMP-Neu5Ac) for *N*-glycosylation, *O*-glycosylation, glycolipids, and *O*-GlcNAc (*O*-linked β-*N*-acetylglucosamine) modification of cytosolic proteins [76].

Glycosylation is a major post-translational mechanism occurring in essentially all mammalian cells. It is characterized by the enzymatic addition of carbohydrate structures (glycans) to secretory and membrane-anchored proteins and lipids in a very well-orchestrated process [8]. Changes in glycosylation are considered a hallmark of cancer, as cancer cells exhibit a completely different repertoire of glycans structures compared with their normal counterparts [8,77]. Glycans have been described to precisely regulate each pathophysiological step of cancer development and progression, from the very beginning of tumour cell dissociation and invasion [77,78,79,80,81], to tumour growth and metastasis. Importantly, glycans was also shown to regulate the insulin/IGF signaling pathway in a cancer context [82], which highlights its importance in the regulatory circuits that integrate metabolic alterations, cancer drug resistance, and cancer cell behaviour.

## 4. Glycosylation as a Regulatory Mechanism of the Insulin/IGF System in Cancer

Changes in the glycosylation machinery occur during the transition from normal to malignant phenotypes giving rise to an increased diversity of glycans structures that are abnormally expressed on the cell surface that further contributes to tumour heterogeneity [8]. Several signaling pathways are known to be dysregulated in a cancer context and some of them have been found to directly impact in the activity of key glycosyltransferases. As example, RAS-RAF-MAPK signaling pathway is frequently upregulated in cancer cells and is particularly involved in the increased expression of the *MGAT5* gene that encodes human *N*-acetylglucosaminyltransferase V (also known as GnT-V) [83]. The increased activity of GnT-V results in the overexpression of β1,6 GlcNAc branching *N*-glycan structures that has been widely associated with malignant and invasive phenotypes [79,84,85,86].

Interestingly, mice with mammary tumours induced by the polyomavirus middle T (PyMT) oncogene (whose expression promotes increased PI3K/AKT1and RAS/MEK/ERK signaling) showed a decrease in tumour growth in *Mgat5*^−/−^ mice [87,88]. Consistently, in the early-stages of these PyMT *Mgat5*^−/−^ mammary tumours they also show lower levels of activation of the PI3K/AKT1signaling [87], and the cell lines derived from the PyMT *Mgat5*^−/−^ are less responsive to insulin-like growth factor (IGF) [89].

The GnT-V-mediated branched *N*-glycans can be further extended giving rise to elongated poly-*N*-acetyllactosamine structures that serve as ligands for galectins, a family of conserved carbohydrate-binding proteins that form galectin-glycan structures on cell surfaces termed “lattices” [90]. The expression of branched *N*-glycans on the extracellular domain of cell surface receptors with a high number of *N-*glycosylation sites, such as on epidermal growth factor receptor (EGFR), insulin-like growth factor receptor (IGFR), and fibroblast growth factor receptor (FGFR), promote the binding to galectins forming the molecular lattice that precludes the endocytosis of glycoprotein receptor, which consequently contributes to signaling activation and increased cell proliferation, tumour growth and oncogenesis [89,91,92].

Another relevant mechanism linking glycans to cancer cell growth occurs during hypoxia. In hypoxic conditions there is inhibition of phosphofructokinase 1 (PFK1, enzyme responsible for the conversion of fructose-6-phosphate to fructose-1,6-bisphosphate-, the first irreversible reaction unique to the glycolytic pathway), which results in *O*-GlcNAcylation at Ser^529^ by OGT (*O*-linked *N-*acetylglucosamine transferase), redirecting the flux of glucose from glycolysis through the PPP and thereby conferring a proliferative advantage to cancer cells [93]. Furthermore, inhibition of *O*-GlcNAcylation at Ser^529^ was found to reduce cancer cell proliferation in vitro and impaired tumour formation in vivo [93].

As a consequence of the shift from oxidative phosphorylation to aerobic glycolysis in cancer cells, the high rates of glucose uptake potentiates the hexosamine biosynthetic pathway (HBP), culminating in the enhancement of metabolic pathways. As a response, the levels of *O*-GlcNAcylation in cancer cells increase, which has a negative impact on cancer cell behaviour [94,95]. *O*-GlcNAc has been described to modulate protein functions by regulating protein phosphorylation and thus affecting key signaling pathways in cancer [96]. Interestingly, the insulin receptor substrate-1 (IRS1) was found to be modified by *O*-GlcNAc, which modulated the effects elicited by insulin and IGF1 [97]. Moreover, the downstream effector AKT1 was also found to be regulated by *O*-GlcNAcylation [98]. Recently, it was demonstrated that high glucose concentration exacerbates colon cancer malignancy by increasing HBP flow, culminating in aberrant glycosylation with increased *O*-GlcNAc levels as well as a tendency to increase levels of branched *N*-glycans [73]. Despite this evidence, the impact of *O*-GlcNAc in the modulation of the insulin/IGF system in cancer remains poorly understood.

Interestingly, evidence points towards the existence of a regulatory circuit between glycosylation, insulin/IGF system and cancer. The interplay between E-cadherin expression—a major tumour suppressor protein in epithelial cancers—and INSR/IGF1R signaling was found to modulate the expression of bisecting *N*-glycans (complex-type *N*-glycan containing bisecting β1,4-linked GlcNAc residue attached to a β-mannose), catalyzed by *N*-acetylglucosaminyltransferase III (GnT-III), encoded by the human *MGAT3* gene [99]. The exogenous expression of E-cadherin in MDA-MB-435 epithelial carcinoma cells (which endogenously lack E-cadherin expression both at mRNA and protein levels) inhibits INSR and IGF1R phosphorylation. The stimulation of MDA-MB-435 + E-cad cells with insulin or IGF1 decreased the bisecting *N*-glycans expression on E-cadherin which consequently up-regulated mesenchymal markers with the enhancement of tumour cell invasion. These observations provide important insights into the effects of insulin/IGF1 signaling in cancer progression through glycosylation modifications [82,84].

**Figure 2:**
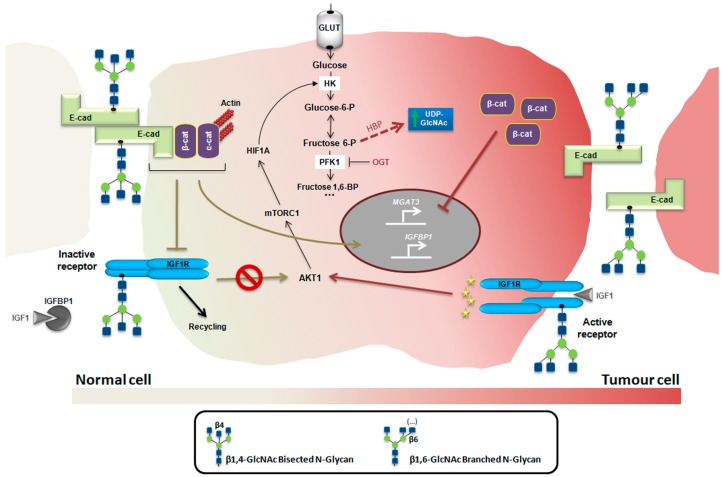
Glycans and insulin/IGF system in cancer: an integrated mechanism. In a normal context, high levels of bisecting GlcNAc *N*-glycans catalyzed by GnT-III favor the establishment of a stable phenotype through E-cadherin-mediated cell-cell adhesion, which in turn promotes *MGAT3* expression establishing thereby a functional feedback loop. Both the stable adherens junctions and IGFBP1 expression lead to the inhibition of IGF1R activity, which were associated with a decrease of the AKT1/mTORC1/HIF1A/HK axis. In a cancer context, the high levels of β1,6-branched *N*-glycans, catalyzed by GnT-V enzyme destabilizes E-cadherin-mediated cell-cell adhesion and favors the activation of IGF1R-mediated signaling thus increasing the AKT1/mTORC1/HIF1A/HK axis. Furthermore, as a consequence of the high HK activity, the hexosamine biosynthetic pathway (HBP) flux become higher, increasing the GlcNAc biosynthesis and the branched *N*-glycosylation. In addition, the translocation of cytoplasmic β-catenin to nucleus promotes inhibition of *MGAT3* expression, that concomitantly with the repression of *IGFBP1*results in a positive feedback mechanism on IGFR1 activity. OGT, *O*-linked *N*-acetylglucosamine transferase. GnT-III catalyzes the bisecting GlcNAc *N*-glycan structure and GnT-V catalyzes the β1,6 GlcNAc branched *N*-glycans.

Taken together, we might be in front of an integrated mechanism in cancer in which the interaction between the insulin/IGF system and metabolic changes might culminate in alterations of the glycosylation of cancer cells that, in turn, fine tune insulin/IGF signaling with major effects on tumour cell development and progression, a biological network that is worth exploring (Figure 2).

Although the evidence presented herein strongly supports the existence of an integrated mechanism between glycosylation modifications and insulin/IGF system in cancer cells, it is worth mentioning that some controversial data exist due to the fact that the effects of *N*-glycans are cell/tissue/organ-specific. As examples, in rat hepatomas the enhanced expression of *MGAT3* (often associated with the suppression of metastasis) has been reported, and the progression of hepatic neoplasms is retarded in mice lacking the bisecting GlcNAc *N*-glycans [100]. Moreover, in bladder cancer the enhanced levels of *MGAT5* (widely associated with malignant and metastatic phenotypes) was associated with low malignant potential and good prognosis [101].

## 5. Conclusions and Future Directions

The insulin/IGF system exhibits a key role in the process of cancer development and progression. Changes in glycans expression also play a fundamental role in the cancer process. We herein propose an integrated mechanism in cancer by which glycans alterations regulate the intricated signaling pathways mediated by the insulin/IGF system with impacts on cancer cell behaviour, cancer cell metabolism, cancer drug resistance and cancer stemness. Targeting this regulatory network in cancer may constitute an interesting approach for novel cancer therapies. The understanding of this network might contribute to finding a way to contradict the biological feedback between glycans and insulin/IGF system, and consequently to control tumour development and progression.

## Abbreviations

Asn	Asparagines
CMP	Cytidine monophosphate
CSC	Cancer stem cells
EMT	Epithelial-mesenchymal transition
GlcNAc	*N*-acetylglucosamine
HBP	Hexosamine biosynthetic pathway
HCC	Hepatocellular carcinoma
NADPH	Reduced nicotinamide adenine dinucleotide phosphate
NSCLC	Non-small cell lung cancer
PPP	Pentose phosphate pathway
PyMT	Polyomavirus middle T
RNA	Ribonucleic acid
RTK	Receptors tyrosine kinase;
Ser	Serine
Thr	Threonine
Tyr	Tyrosine
UDP	Uridine diphosphate
XIAP	Increasing x-linked inhibitor of apoptosis protein
